# Injury and training history are associated with glenohumeral internal rotation deficit in youth tennis athletes

**DOI:** 10.1186/s12891-020-03571-0

**Published:** 2020-08-15

**Authors:** Kristin Kalo, Lutz Vogt, Johanna Sieland, Winfried Banzer, Daniel Niederer

**Affiliations:** 1 Department of Sports Medicine and Exercise Physiology, Goethe University Frankfurt, Ginnheimer Landstraße 39, 60487 Frankfurt am Main, Germany; 2 Department of Preventive and Sports Medicine, Institute of Occupational, Social and Environmental Medicine, Goethe University Frankfurt, Frankfurt am Main, Germany

**Keywords:** GIRD, Shoulder injury, Tennis player, Training history

## Abstract

A glenohumeral internal rotation deficit (GIRD) of the shoulder, is associated with an increased risk of shoulder injuries in tennis athletes. The aim of the present study was to reveal the impact of 1) age, sex, specific training data (i.e. training volume, years of tennis practice, years of competitive play) and 2) upper extremity injuries on GIRD in youth competitive tennis athletes.

A cross-sectional retrospective study design was adopted. Youth tennis players (*n* = 27, 12.6 ± 1.80 yrs., 18 male) belonging to an elite tennis squad were included. After documenting the independent variables (anthropometric data, tennis specific data and history of injury), the players were tested for internal (IR) and external (ER) shoulder rotation range of motion (RoM, [°]). From these raw values, the GIRD parameters ER/IR ratio and side differences and TRoM side differences were calculated. Pearson’s correlation analyses were performed to find potential associations of the independent variables with the GIRD outcomes.

A significant positive linear correlation between the years of tennis training and IR side asymmetry occurred (*p* < .05). A significant negative linear relation between the years of tennis training and the ratio of ER to IR range of motion (RoM) in the dominant side (*p* < .05) was found. The analysis of covariance showed a significant influence of the history of injuries on IR RoM (*p* < .05).

Injury and training history but not age or training volume may impact on glenohumeral internal rotation deficit in youth tennis athletes. We showed that GIRD in the dominant side in youth tennis players is progressive with increasing years of tennis practice and independent of years of practice associated with the history of injuries. Early detection of decreased glenohumeral RoM (specifically IR), as well as injury prevention training programs, may be useful to reduce GIRD and its negative consequences.

## Background

Shoulder injuries are the most common upper limb injuries in professional tennis athletes [1]. Especially youth athletes show chronic overuse disorders and a higher risk for acute injury as possible implication of repetitive stress on the glenohumeral joint during growing periods [2]. The numerous serves throughout a tennis match can cause recurrent microtrauma and may lead to physiological adaptations of the joint as well as of the joint’s surrounding soft tissue [3, 4]. A permanent tightness of the posterior rotator cuff, muscles and tendons, therefore, lead to alterations in scapular and humeral kinematics and in a stable change in shoulder motion of tennis athletes [5–7]. More precise, these physiological adaptations often result in a decreased internal rotation (IR) range of motion (RoM) of the shoulder joint combined with a decreased total range of motion (TRoM) of the dominant compared to the non-dominant limb [8]. This glenohumeral internal rotation deficit (GIRD), however, is associated with an increased risk for shoulder injuries [9–13] and might have an impact on the performance of youth tennis players [14]. An increased shoulder RoM may be, contrarily, protective against injuries [2]. Current literature differentiate between an anatomical GIRD and a pathological GIRD. Most commonly, an anatomical GIRD is defined as a loss of IR greater than 18°-20° compared to the contralateral shoulder [15]. In addition, Rose and Noonan [16] defined a pathological GIRD as the loss of glenohumeral IR combined with a loss in TRoM greater than 5 degrees.

Various hints of the relationship between GIRD and upper extremity injuries in different throwing activities are available [17–19]. None of these studies, nevertheless, examined this association in tennis athletes. Beyond that, the association of GIRD and shoulder injuries is less clear for adolescent athletes [20]. Moreover, the factors fostering decreased glenohumeral joint motion in youth tennis players are still unclear. Previous studies indicated that a loss of IR RoM in the dominant shoulder may be linked to the player’s age, his/her years of tennis practice and years of professional play, but the results did not reach statistical significance [21, 22]. The influence of sex on GIRD is yet unclear [22]. A better understanding of those predictors might be helpful to prevent GIRD and its potential consequences as well as to emphasize corresponding treatment methods.

On the basis of these research deficits, the aims of this study were to 1) gain further evidence on the potential associations of age, sex, specific training data (i.e. training volume, years of tennis practice, years of competitive play), bilateral passive shoulder IR and ER RoM as well as the occurrence of GIRD and 2) reveal potential associations of upper extremity injuries and IR RoM/GIRD under consideration of relevant surrogates in youth competitive tennis athletes. We hypothesize that 1) with increasing age, training volume, years of tennis practice as well as years of professional play, the glenohumeral IR RoM decreases and that 2) the history of injury as well as players’ sex impact this relation.

## Methods

### Study design and ethics

A cross-sectional retrospective study design was adopted. All players underwent a sports medical examination as well as tennis specific functional diagnostics. The measurement data were used for reviewing health status and for individual training adjustments. The study was performed in accordance with the declaration of Helsinki. Each athlete and his/her legal representative signed informed consent.

### Sample

Youth tennis athletes belonging to a regional elite tennis squad actively competing on national or international level were included. Exclusion criteria consisted of (current) delayed onset muscle soreness, upper extremity injuries and shoulder pain in the previous 3 months, shoulder or elbow surgery in the previous 12 months, and analgesic consumption in the past 48 h. A total of 27 players aged between 10 and 17 years (12.6 ± 1.8 yrs., 18 male) subscribed informed consent after screening for inclusion and exclusion criteria. No athlete had to be excluded, no athlete or his/her legal representative withdrew informed consent.

### Measurements

All participants underwent a sports medical examination, followed by anthropometrics and tennis specific data i.e. (1) training volume (playtime in hours per week), (2) years of tennis practice, (3) years of competitive play (tennis league), and (4) history of injury assessments (documented via questionnaire and by means of a structured interview, injury was defined according to the time loss concept). Afterwards, shoulder RoMs were measured.

The shoulder mobility consisting of IR and ER RoM was measured in supine position on a physiotherapy bench. The shoulder was held by an examiner at 90° abduction with 90° flexion in the elbow. A second examiner placed a clinical goniometer (MIE Medical Research Ltd., Leeds, UK) in the mid-point of the distal end of the vertically held forearm (neutral position). The goniometer was found to be a reliable tool in quantifying shoulder RoM (ICC: > 0.78) [23, 24]. The first examiner internally rotated the glenohumeral joint while stabilizing the scapula on the bench to avoid compensatory movements. When the scapula began to move into protraction or anterior tilt, the measurement endpoint was reached and the respective RoM value was noted. After returning to the starting position, the arm was externally rotated using the same procedure. The order of shoulders to be assessed was randomized. The assessment was performed by the same examiner (sports therapist).

### Data processing and statistics

To calculate TRoM, IR and ER RoM were summed. Absolute (°) and relative (%) differences/asymmetries between the non-dominant and the dominant shoulder were calculated for TRoM, IR and ER RoM. The ER/IR ratio was calculated by subtracting the RoMs for ER from IR for each limb side. The IR of all players was further subdivided into “no pathological GIRD” (IR deficit) and “pathological GIRD”. As previously described by Rose and Noonan [16], we define a pathological GIRD, based on a conservative estimation, as an IR deficit of > 20° with a loss in TROM of > 5° when compared to the contralateral shoulder.

Data distribution was evaluated using Kolmogorov-Smirnov statistics with Lilliefors correction. Descriptive statistics (means ± standard deviations plus range, only in case of normal distribution of the data) for each of the variables were calculated. Depending on the distribution, Pearson’s correlation or Spearman’s rank correlation to determine potential relationships between players’ age, training volume (i.e., hours per week), years of tennis practice as well as years of competitive play with all aspects of GIRD (IR, ER RoM, TRoM, ER/IR ratio) were calculated. For absolute values of R^2^, 0–0.19 is regarded as very weak, 0.2–0.39 as weak, 0.40–0.59 as moderate, 0.6–0.79 as strong and 0.8–1 as very strong correlation. Afterwards, all significant predictors for GIRD were selected as co-factors. Analysis of co-variance followed to investigate potential differences in all aspects of shoulder motion depending on sex and history of injury including the a priori selected covariates into the model. All analyses were performed using SPSS version 24 (SPSS inc., Chicago, USA) with a significance level a priori set at α = .05.

## Results

Overall, 24 players were right-hand dominant, three left-handed. No athlete trained forehand strokes with both the dominant and non-dominant arm (no crossover effect). Two players had a history of upper extremity injuries at their dominant side, one athlete showed an injury in the non-dominant arm (1x bursitis, 1x fracture of collarbone, 1x impingement) and three showed bilateral orthopedic abnormalities in their shoulder girdle (2x misalignment of the shoulders, 1x Scapula alata) prior to those 3 months. The training volume was (mean) 7.1 (±(standard deviation) 2.6, (range) 4–16) hours per week of tennis play and an additional strengthening and stretching program of 3.3 (±1.0, 1–5) hours per week. The athletes played tennis for 5.9 (±2.3, 2–10) years and competitive tennis in the elite squad for 3.3 (±1.6, 1–6) years. The players showed an IR RoM of 76.5 (±15.5) degrees in their dominant side and of 83.6 (±15.6) degrees in their non-dominant side. The ER RoM was 98.9 (±11.8) degrees in the dominant shoulder and 98.4 (±12.6) degrees in the non-dominant shoulder. The TRoM was 175.4 (±19.0) degrees in the dominant and 182.0 (±17.0) degrees in the non-dominant side. Five athletes showed a pathological GIRD. Table 1 shows the anthropometric data, training related data and upper extremity injury data for players with and without pathological GIRD.

**Table 1:** Anthropometric, training related and upper extremity injury data for players with and without pathological GIRD. Data are displayed as mean ± standard deviation and range, if not stated otherwise

	No pathological GIRD	Pathological GIRD
**Sex distribution [n]**	female: *n* = 7 male: *n* = 15	female: *n* = 2 male: *n* = 3
**Preferred hand [n]**	right: *n* = 20 left: n = 2	right: *n* = 4 left: *n* = 1
**Body height [cm]**	163.6 ± 11.9 146.0–181.5	159.4 ± 12.7 152.0–182.0
**Body weight [kg]**	51.1 ± 12.1 33.0–69.5	47.0 ± 13.4 40.0–71.0
**Age [years]**	12.6 ± 1.9 10–17	12.2 ± 1.6 11–15
**BMI [kg/m**^**2**^**]**	18.7 ± 2.2 14.7–21.9	18.2 ± 1.8 17.2–21.4
**Training volume [hours/week]**	6.8 ± 1.9 4–11	8.3 ± 4.4 5–16
**Years of tennis practice [years]**	6.1 ± 2.3 2–10	5.3 ± 2.6 3–9
**Years of competitive play [years]**	2.7 ± 1.9 0–6	3.2 ± 1.9 0–5
**Number of injuries per extremity [n]**	Dominant side: 2 Non-dominant side: 1 Bilaterally: 3	Dominant side: 0 Non-dominant side: 0 Bilaterally: 0

The differences in shoulder motion compared bilaterally (i.e. relative IR RoM, absolute IR RoM, ER/IR ratio) for all athletes, such with a GIRD and such without are shown in Table 2. No athlete reported pain during the RoM measurement.

**Table 2:** Shoulder RoM with and without GIRD. Data are displayed as mean ± standard deviation and range, if not stated otherwise. Differences of dominant compared to non-dominant arm

	All athletes	No pathological GIRD athletes	Pathological GIRD athletes
**Relative IR side asymmetry/ratio (%)**	0.9 ± 0.2 0.5 – 1.4	1.0 ± 0.2 0.8 – 1.4	0.7 ± 0.1 0.5 – 0.8
**Absolute IR side asymmetry/ratio (°)**	− 7.1 ± 19.5 − 65.0 - 30.0	−0.4 ± 12.9 − 17.0 - 30.0	−36.4 ± 17.1 − 65.0 - (-20.0)
**ER/IR ratio**	1.1 ± 0.2 0.7 – 1.5	1.0 ± 0.2 0.7 – 1.5	1.4 ± 0.2 1.2 – 1.5

**Fig. 1:**
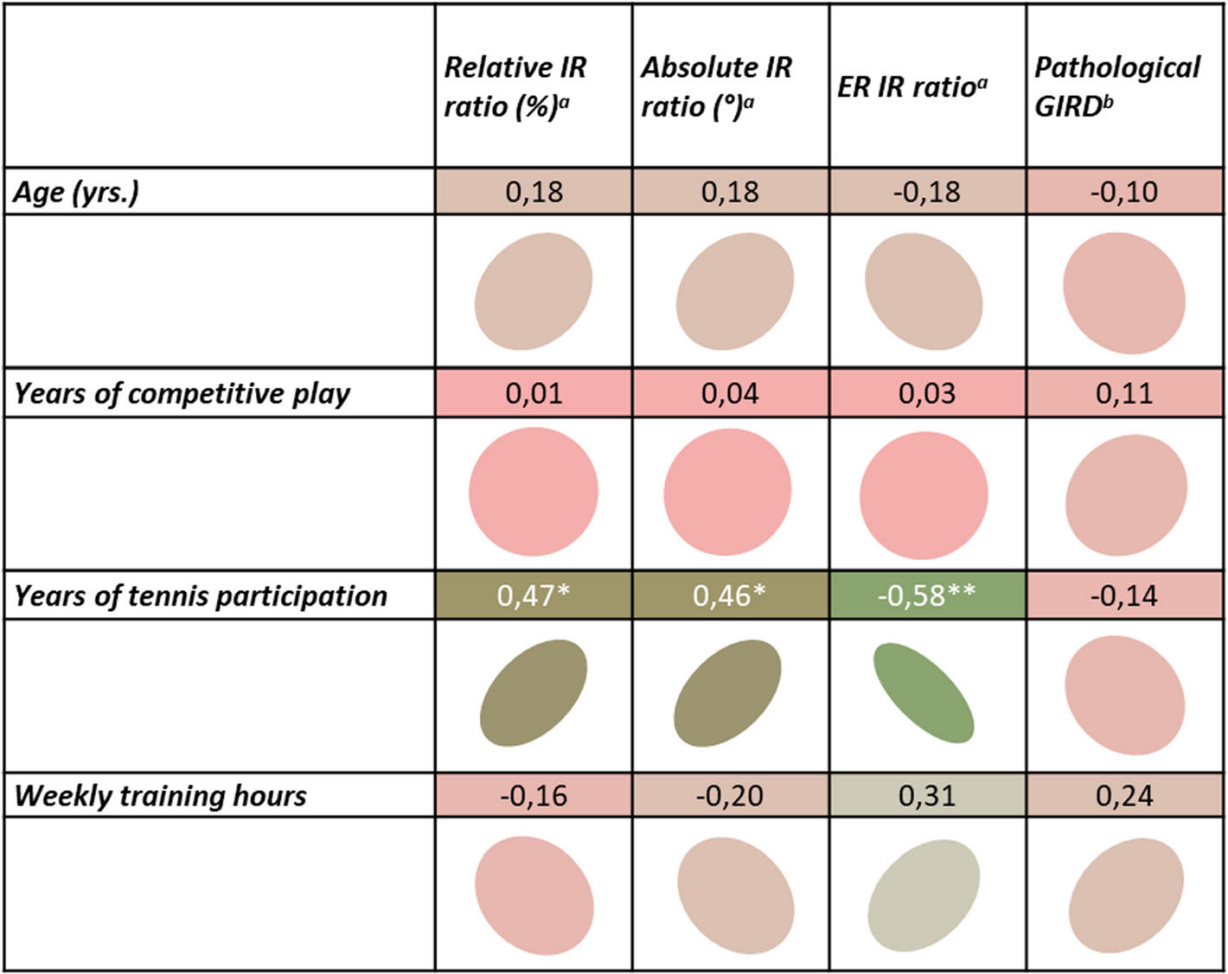
Correlation matrix. Strong correlations are displayed in green and weak correlations in red. The form of the circle depicts the variance explanation; the slope highlights the direction of effects. Colour intensity and the form of the circle are proportional to the correlation coefficients. ^a^ dominant to non-dominant difference; ^b^ clinical relevance of a GIRD; *correlation is significant at the .05 level; ** correlation is significant at the .01 level

Figure 1 shows the results of the correlation analyses. All *p*-values and the types of correlation as well as the correlation coefficients are displayed.

The results showed a positive correlation between years of tennis training and the ratio of relative IR side-to-side differences. The absolute IR RoM differences correlated with the years of tennis practice. A negative relation occurred between years of tennis training and the ratio of ER to IR RoM. There was no correlation between subdivisions of GIRD and years of tennis practice. Age, training volume and years of competitive play had no influence on changes in shoulder motion.

Conclusively, only years of tennis practice was associated with a (non-pathological) GIRD. The subsequently performed analysis of covariance (ANCOVA, years of tennis practice as co-factor) showed a significant influence of the history of injuries on relative (R^2^ = .58, F = 9.7, *p* < .05) and absolute IR RoM values when compared bilaterally (R^2^ = .46, F = 11.1, *p* < .05), but not for ER/IR RoM ratio (*p* > .05) or for the existence of a pathologic GIRD (*p* > .05). Conclusively, a history of injury was associated, still after the adjustment for training years, with (non-pathological) GIRD. Sex had no influence on any variable (*p* > .05).

## Discussion

### Hypothesis testing and comparison with existing evidence

The results showed an increase in side-to-side shoulder IR RoM difference and a decrease in the ER/IR RoM ratio with increasing years of tennis training but not with age, years of competitive play, or training volume (hours per week). The years of tennis practice had no influence on the occurrence of pathological GIRD. Our results are mostly in line with previous research. Moreno-Pérez et al. [21] investigated whether professional tennis players aged 14–21 years with a history of self-reported shoulder pain showed differences in TRoM between the dominant and non-dominant shoulder compared to asymptomatic controls. The authors revealed a negative correlation between TRoM and age as well as between TRoM and years of total play. Nevertheless, a statistically significant influence of age, years of tennis practice or years of professional play on internal rotation adaptations was not found. These findings are consistent with previous results from Kibler et al. [22], who examined professional tennis players aged 14 to 21 years. In contrast to that, our study showed a significant influence of years of tennis practice on IR RoM. It can conclusively be assumed that changes in rotational shoulder RoM are not dependent on age, but on years of tennis practice. A reason of the non-association of the occurrence of pathological GIRD and the years of playing tennis may be found in the low number of athletes suffering from pathological GIRD in our study. However, non-pathological GIRD often leads to pathological changes in the shoulder joint and can therefore be seen as a lead to a pathological GIRD [16]. It is thus important to screen for and subsequently treat a non-pathological GIRD.

We further showed that, after controlling for the years of tennis practice, the history of injuries but not sex was associated with the occurrence of a GIRD and a reduction in IR RoM. Although a number of trials imply a relationship between upper extremity injury and GIRD in overhead athletes, the associations in adolescent athletes are less clear [20]. Moreover, this is the first study to examine a possible influence of injuries in youth tennis athletes. Our results comply with previous research, which stated that RoM deficits are more common in overhead athletes who have previously been injured [20]. Moreover, some research showed a positive relationship between posterior capsule thickness and scapular upward rotation as well as between abnormal scapular positioning and GIRD. The scapula acts, especially during overhead throwing, as an important link between the humerus and trunk. It allows for an immense degree of freedom at the glenohumeral joint [5, 25]. However, a loss of normal scapular orientation, lead to a change in shoulder motion and joint kinematics as potential sources of shoulder injuries.

One may assume that the more years an athlete participates in training sessions, the higher the chance of anatomical adaptation occuring, may ultimately lead to injuries. Yet, this association persists even after having eliminated the impact of training years. Thus, one may speculate that both injuries and/or years of training are independent risk factors for the development of GIRD.

### Clinical implications

In accordance with the relevant reference values for youth tennis athletes [26], our sample showed lower total and IR RoM in their dominant compared to the non-dominant side. Even though the RoM in our sample was slightly above the age-matched reference average, the athletes included in the present study can be considered as representative for the underlying youth tennis athletes’ population [26].

Taking into account that GIRD increases with the number of years of tennis training and that it is influenced by injuries, it is important to prevent a change in shoulder mobility, especially in the early days of an athlete’s career or of aiming-at-being-professional youth athletes. There is evidence that altered shoulder mobility can be easily treated [27, 28]. Established conservative treatments adopt stretching [27], mobilization techniques [29] and strengthening of the shoulder girdle [30]. These strategies can be applied preventive in addition to the general tennis training and may be applied before the first onset of a (non- or pre-pathological) GIRD. Previous research suggest that a stretching program of the posterior capsule and cuff can help improve and restore normal IR RoM [37, 38]. Moreover, there are indications of acute effects of tennis practice sessions on the shoulder mobility [13]. The authors highlighted that stretching the shoulder girdle directly after training or competition may prevent physiological adaptations to stress in the shoulder and therefore the development of GIRD. However, some studies claim, that stretching directly after exercising or to intense stretching might even produce delayed onset muscle soreness or subsequent inflammation to the joint surrounding tissue [31, 32]. Further investigations on such acute treatment effects and appropriate recovery strategies after training or competition are still necessary.

It is still unclear whether physiological shoulder adaptations in overhead athletes are protective against tissue damage or if previous tissue damage leads to future shoulder injuries [20]. There is, furthermore, a debate whether physiological GIRD may even improves athletes’ performance instead of being a risk factor for injuries [33]. Manske et al. [12] stated that a GIRD may be required to generate sufficient forces during tennis serves and does not lead to a pathological GIRD. An increased load on the posterior capsule may lead to a greater force for the serve velocity as well as a greater risk in shoulder injury [34]. In contrast, GIRD caused by posterior capsule tightness is associated with a humeral retroversion and scapular dyskinesia. The resulting shift in the arthrokinematics of the glenohumeral joint is associated with the onset of rotator cuff tears, internal impingement, and superior labrum anteroposterior lesions [25]. Moreover, reduced shoulder mobility may have an impact on other parts of the body, e.g. the back or hip, which have to compensate the decreased mobility in the shoulder girdle [6, 35]. Scher et al. [36] detected a relationship between dominant hip extension and dominant shoulder ER RoM in both pitchers and nonpitchers with a history of shoulder injury and between dominant hip extension and dominant shoulder IR in nonpitchers with no history of shoulder injury. Restrictions in the shoulder RoM may therefore increase mechanical demands on other body parts and joints that lead to further injury. Further studies are needed to examine the associations and effects of a GIRD (treated, prevented or untreated) and in respect of the whole body.

When assuming that the years of tennis practice is a determining factor for developing a GIRD in the dominant side, and that a physiological GIRD is a predictive factor for a pathological GIRD [16] it would be beneficial to treat anatomical adaptations in early years of training before any flexibility restrictions occur. Against the background of the facts that an ER or TRoM deficit can be decisive for shoulder injuries, further high quality and long-term studies should investigate the role of GIRD in increasing the risk of injury. Considering all that, it is important to identify a GIRD by corresponding screening methods. Even so, since stretching is easy, safe and cost-effective, athletes without a measured deficit should regularly stretch if continuous tennis training is to take place.

### Methodological considerations and limitations

We have only assessed associations of potential risk factors, injuries and GIRD and have not analysed potential aetiologically relevant mechanisms like imbalances in strength, scapular dyskinesis, or a tightness of the posterior capsule. Further research should take these potential pathophysiological causes into account. Additional research is needed to prospectively investigate the influence of GIRD and its causes on shoulder injuries and tennis performance in players of all ages and levels.

## Conclusion

A loss of glenohumeral internal rotation on the dominant side in youth tennis players is given. This loss is progressive with increasing years of tennis practice and independently associated with the history of injuries in the dominant side. It can be assumed that changes in rotational shoulder RoM are not dependent on age, but on years of tennis practice. This phenomenon was found to the same extent in both boys and girls. Primary preventive programs, early detection of a decreased glenohumeral RoM (in particular IR), as well as secondary injury prevention training programs may be useful. However, additional studies are required to further understand the relationship between rotation deficit, specific training data and risk of shoulder injury.

## Data Availability

The datasets used and/or analysed during the current study available from the corresponding author on reasonable request.

## Abbreviations

GIRD: Glenohumeral internal rotation deficit
IR: Internal rotation
ER: External rotation
RoM: Range of motion
